# Reliability and validity of methods in the assessment of cold-induced shivering thermogenesis

**DOI:** 10.1007/s00421-019-04288-2

**Published:** 2020-01-18

**Authors:** Josh T. Arnold, Zach Hemsley, Simon G. Hodder, George Havenith, Alex B. Lloyd

**Affiliations:** Environmental Ergonomics Research Centre, Loughborough University, Leicestershire, LE11 3TU UK

**Keywords:** Cold, Shivering, Electromyography, Oxygen uptake, Mechanomyography

## Abstract

**Purpose:**

To compare two analytical methods for the estimation of the shivering onset inflection point, segmental regression and visual inspection of data, and to assess the test–retest reliability and validity of four metrics of shivering measurement; oxygen uptake (V̇O_2_), electromyography (EMG), mechanomyography (MMG) and bedside shivering assessment scale (BSAS).

**Methods:**

Ten volunteers attended three identical experimental sessions involving passive deep-body cooling via cold water immersion at 10 °C. V̇O_2_, EMG, and MMG were continuously assessed, while the time elapsed at each BSAS stage was recorded. Metrics were graphed as a function of time and rectal temperature (*T*_re_). Inflection points for intermittent and constant shivering were visually identified for every graph and compared to segmental regression.

**Results:**

Excellent agreement was seen between segmental regression and visual inspection (ICC, 0.92). All measurement metrics presented good-to-excellent test–retest reliability (ICC’s > 0.75 and 0.90 respectively), with the exception of visual identification of intermittent shivering for V̇O_2_ measurement (ICC, 0.73) and segmental regression for EMG measurement (ICC, 0.74). In the assessment of signal-to-noise ratio (SNR), EMG showed the largest SNR at the point of shivering onset followed by MMG and finally V̇O_2_.

**Conclusions:**

Segmental regression provides a successful analytical method for identifying shivering onset. Good-to-excellent reliability can be seen across V̇O_2_, EMG, MMG, and BSAS, yet given the observed lag times, SNRs, along with known advantages/disadvantaged of each metric, it is recommended that no single metric is used in isolation. An integrative, real-time measure of shivering is proposed.

## Introduction

Shivering can be defined as the ‘simultaneous asynchronous contraction of the muscle fibres in both flexor and extensor muscles in response to a cold stressor’ (Bligh 1985). Involuntary contraction of skeletal muscle during shivering serves exclusively to produce heat (thermogenesis), with little of the energy expended as physical work. Both the onset and magnitude of shivering are influenced by corresponding reductions in skin or deep body temperature, but also a range of non-thermal factors (Kenny et al. 1998; Mekjavic and Eiken 2006). Initial reductions in skin temperature are believed to predominantly drive mild intermittent shivering, while further reductions in both core and skin temperature are believed to drive continuous intense shivering thermogenesis (Stocks et al. 2004; Haman and Blondin 2017). Peak shivering in adults can elicit heat production up to six times basal metabolic rate (Iampietro et al. 1960; Glickman et al. 1967; Eyolfson et al. 2001). Concurrent vasoconstriction and piloerection also plays a role in the preservation of heat, thus enhancing the effectiveness of the shivering thermogenesis (Burton and Edholm 1955; Parsons 2014). Shivering and/or the associated heat production can be quantified via several metrics; objectively via whole-body oxygen uptake (V̇O_2_), electromyography (EMG) or mechanomyography (MMG), also subjectively via the bedside shivering assessment scale (BSAS) (Badjatia et al. 2008).

### Whole-body oxygen uptake (V̇O_2_)

V̇O_2_ can be used to estimate energy expenditure associated with thermogenesis through the metabolism of fats and carbohydrates. Thus, disregarding the relatively low contribution of non-shivering thermogenesis to total heat production in the cold—indeed brown adipose tissue has shown to contribute ~ 1% to the cold-induced thermogenesis associated elevation in energy expenditure (Din et al. 2016)—V̇O_2_ can be used to provide a proxy for the measurement of shivering. V̇O_2_ has been used widely across shivering research, including efforts to determine peak shivering intensity, which can reach 40% V̇O_2_max in some individuals (Eyolfson et al. 2001). Methodologically, V̇O_2_ provides a holistic whole-body representation of metabolism, including basal metabolic rate and work across multiple muscle groups with varying degrees of tone and dynamic contraction, both thermogenic and behavioral or locomotive in purpose. As such, assumptions must be made with regards to the measurement of shivering through elevated V̇O_2_, further constrained by the potential temporal delay in the signal relative to actual shivering onset.

### Electromyography (EMG)

Assessment of the electrophysiological signals generated by shivering muscles is well established (Bell et al. 1992), including sophisticated spectral analysis of EMG patterns to quantify muscle fibre recruitment and substrate fuel selection during shivering (Weber and Haman 2005). Contractile forces generated during sustained whole-body shivering may equate to approximately 15–20% maximal voluntary muscular activation, as estimated via EMG (Stocks et al. 2004). It has been suggested that with an appropriately placed surface probe, EMG provides a well-defined instantaneous signal, which can identify shivering onset prior to visual detection (Hemingway 1963). Nonetheless, although prominent muscles involved in shivering thermogenesis have been identified [i.e. pectoralis major, trapezius, sternocleidomastoid and deltoideus (Blondin et al. 2015)], variation exists between individuals in the order of recruited muscles during mild shivering (Haman and Blondin 2017). Indeed, EMG signal is specific to the muscle(s) which lay directly beneath the sensor; as such, difficulty is often encountered in the quantification of more subtle shivering responses, where the activation signals of muscles which are not in close proximity to the sensor may be missed (Haman and Blondin 2017). Surface EMG also provides no indication of the activity of deeper muscle groups, which also contributes to shivering thermogenesis (Blondin et al. 2015; Din et al. 2016).

### Mechanomyography (MMG)

Shivering presents itself in the form of a muscular ‘tremor’, thus mechanical quantification of shivering magnitude and frequency is possible using three-axis accelerometry, a form of mechanomyography (MMG). The use of such mechanical methods appears less common in shivering focused research (McKay et al. 2010, 2013), yet the benefits and limitations of accelerometry closely match that of EMG, i.e. an instantaneous signal artifact and clear signal-to-noise ratio, restricted by the specific placement of the sensor over the single or localized group of activated muscles. It is possible that a high-quality accelerometer may provide a more sensitive measure of muscular activity than EMG for shivering thermogenesis, since the electrical activity of the muscle during extremely mild shivering lies close to the lower limit of resolution of surface EMG. Indeed, with only 1–2% of resting muscle motor units active in muscular ‘microvibrations’, EMG tracings show infrequent spikes, while mechanical activity may be more reliably detected with the high sensitivity and low thresholds of accelerometry (McKay et al. 2013). Furthermore, it is conceivable that MMG suffers less extraneous noise than EMG, thus further improving the signal–noise ratio, subsequently aiding analysis of shivering thresholds.

### Bedside shivering assessment scale (BSAS).

Quantifying shivering via visual identification and palpation, the BSAS offers a fast and convenient 4-point scale, allowing broad differentiation of graded metabolic responses to shivering thermogenesis (Badjatia et al. 2008). Commonly used within clinical practice, previous research has identified adequate inter-rater reliability of the BSAS between observers, along with moderate correlations of BSAS against EMG and V̇O_2_ in shivering intensity (Badjatia et al. 2008; May et al. 2011; Olson et al. 2013). The capacity of BSAS to track dynamic changes in shivering intensity, along with the agreement between BSAS and objective metrics in defining shivering onset remains unclear.

### Quantifying shivering thresholds

In addition to the listed metrics used to measure shivering activity, various analytical methods exist for the quantification of the shivering onset threshold, also referred to as the inflection point or break point. Previous thermoregulatory research has validated the use of segmental linear regression for the objective identification of the sweating threshold, fitting two intersecting regression lines using a least-squares fit (Cheuvront et al. 2009). Recent shivering research has also adopted this form of analytical assessment (Fujimoto et al. 2017). Though segmental regression provides a convenient tool for use in purely autonomic sweating research, its use in shivering research has yet to be validated. Indeed, numerous factors such as voluntary muscular movement, increased muscular tone, voluntary suppression of shivering and inconsistencies in burst shivering patterns, often confound the presence of a clearly identifiable inflection point. Whether visual identification of inflection points provides an improved method for extracting threshold information in the presence of such factors has yet to be determined.

Despite the use of various forms of shivering measurement and threshold quantification in research and clinical practice, a direct comparison between metrics and methods has not been performed. This may be of importance in light of research highlighting the potential of various secondary and non-thermal factors to bring both forward or delay the onset of shivering (Castellani et al. 1985; Kenny et al. 1998; Fujimoto et al. 2019). Additionally, most studies have used one or possibly two measures of shivering onset (Cabanac and Massonnet 1977; Mittleman and Mekjavic, 1991; Cheng et al. 1995; Haman et al. 2004; Blondin et al. 2015; Fujimoto et al. 2019), while no study has developed an integrative measure of shivering onset, which combines multiple modalities of shivering measurement into a variable that is reflective of shivering onset and severity.

The aim of this methodological study was twofold: to compare the validity of two independent analytical methods for the quantification of the shivering inflection point, segmental regression, and visual identification of data. Second, to assess and compare the test–retest reliability and validity of four independent metrics for the assessment of shivering onset, V̇O_2_, EMG, MMG, and BSAS. A subsample of the data herein was presented at the 7th International Conference on the Physiology and Pharmacology of Temperature Regulation (Arnold et al. 2018).

## Methods

### Participants

Ten healthy volunteers, eight males and two females (age 23 ± 3 years; stature 1.75 ± 0.07 m; body mass 71.1 ± 11.5 kg; BMI 23 ± 2 kg/m^2^) were recruited from the student population of Loughborough, UK, between February and April 2018. All participants were physically active, non-smoking individuals, over 18 years of age. Exclusion criteria included smokers and any individuals with a history of muscular, neurological, or cardiovascular debilities. Participants provided written informed consent. Ethical approval was granted by the Ethics Approvals Committee at Loughborough University and the research was conducted in accordance with the Declaration of Helsinki, 2008.

### Study design

**Fig. 1:**
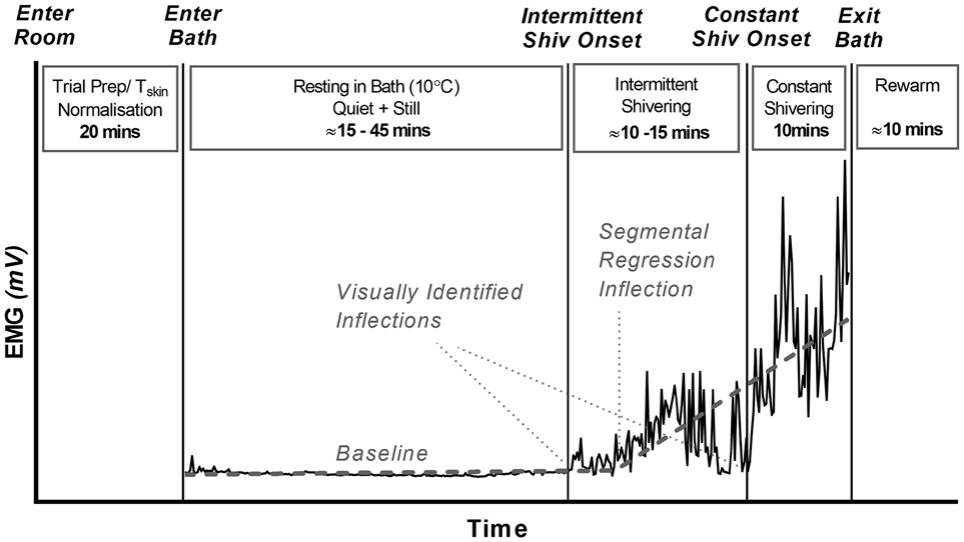
Schematic representation of the methodological procedures with sample EMG output. Repeated measures design in which ten participants visited the laboratory on three occasions undertaking three identical experimental sessions. Shivering onset defined by visual inspection and segmental linear regression (least-squares fit)

Utilizing a repeated measures design, participants visited the laboratory on three occasions undertaking three identical experimental sessions (Fig. 1). Sessions were undertaken at the same time each day to exclude the impact of the circadian rhythms upon thermoregulation, as observed in previous research (Kondo et al. 2009). Consecutive visits to the laboratory were also confined to a consecutive 3–5 day period for each participant to minimize the impact of extraneous lifestyle factors, particularly the menstrual cycle in female participants. To elicit deep-body cooling, experimental trials involved passive cooling via lower body cold water immersion, undertaken in an air-conditioned room. Participants adopted a semi-recumbent seated position in a bathtub, with cold water immersion (~ 100 L) to the mid torso and arms rested on the sides. Environmental conditions were prepared and maintained within a strict range across all trials (bath temperature, 10.2 ± 0.4 °C; ambient air temperature, 21.2 ± 1.2 °C; relative humidity, 19 ± 4%). Bath temperature set point was maintained using an electrically isolated 4 kW chiller unit (TAEevo M10, ICS Cool Energy, UK) and a low-pressure water pump (50 l/min) was connected to heat exchangers to ensure adequate circulation of water. In addition to cold water immersion, an industrial fan (650 mm) was placed 1.5 m away from the participant to encourage convective cooling of the upper body (air velocity, 3.5 ± 0.18 m·s). Neoprene wetsuit boots (5 mm) were worn during cold water exposure to protect the feet from extreme discomfort and reduce the risk of non-freezing cold injury. Upon completion of each experimental trial, participants were carefully rewarmed. The lower legs were first towel dried, all physiological apparatus were removed before the participant was offered a warm shower to restore thermal comfort and encouraged to actively rewarm (exercise) if possible. Monitoring of *T*_re_ continued to be until it returned to within 0.5 °C of the pre-cooling value.

### Procedure

One week prior to the first visit, participants were briefed with regards to the study aims and asked to refrain from alcohol, caffeine, and any non-routine vigorous activity 24 h preceding each session. Participants were also asked to best replicate exactly what they had eaten in the 24 h prior to the first session across the subsequent sessions. On arrival to the laboratory, mass and stature were first assessed. Participants were provided with a rectal thermistor (400-AC Temperature Probe, Viamed Ltd., UK) and instructed to self-administer 100 mm beyond the anal sphincter before entering the test environment (European Committee for Standardization 2004). Participants then remained seated in a thermoneutral environment, wearing swim shorts with the addition of a sports bra for females, for a standardized 20-min period, allowing skin temperature to normalize and the attachment of all necessary sensors. Immediately following, participants entered the bath and data collection commenced. Participants were encouraged to relax, remain still, and donned a set of ear defenders to limit any noise distractions or time cues, thus the potential for accidental voluntary contributions to muscular activity. Oxygen uptake, EMG, and MMG were continuously assessed throughout the trial, while the time elapsed relative to various BSAS stages (see below) was noted down. Participants remained in the bath until 10 min after they were deemed to be continuously whole-body shivering via visual assessment, at which point the test ended. Core temperature was continuously tracked and assessed via rectal temperature (*T*_re_) for the duration of the trial and rewarming period. A *T*_re_ cut-off limit was maintained at 35.0 °C across all trials at which point any given trial would be terminated.

### Measures of shivering

Four independent metrics were compared as measures for the assessment of the shivering response; bedside shivering assessment scale (BSAS), whole-body oxygen uptake (V̇O_2_), electromyography (EMG), and mechanomyography (MMG). Bedside shivering assessment scale (Badjatia et al. 2008) via visual subjective rating was administered by a member of the research team (ZH) and validated by a second member of the research team (JA) across a subsample of ten trials to ensure agreement. Three distinct stages were noted, modified from the original BSAS to better align with objective metrics; *BSAS 1* minor shivering-related muscular twitches, *BSAS 2* noticeable intermittent burst shivering of the extremities, *BSAS 3* generalized and sustained whole-body shivering. Continuous breath-by-breath V̇O_2_ was assessed via a metabolic cart (Metamax 3B, Cortex, Germany) and analyzed in Metasoft® (Cortex, Germany) with a two-point calibration prior to each trial. Electromyography assessed via wireless surface EMG, with probes (DataLITE Wireless, Biometrics Ltd, UK; probe mass, 17 g) placed on the right hand side of the body, over the center of the muscle belly at the pectoralis major, deltoid, and trapezius using double-sided adhesive medical tape. Raw EMG was sampled at 1000 Hz with a gain of 1000 and the signal rectified in DataLITE^©^, filtered to remove spectral components at 50 Hz, and the related harmonics. No attempt was made to normalize EMG data against a maximal voluntary contraction, as the assessment of relative shivering intensity resides outside the scope of this paper. Mechanomyography assessed via custom-built tri-axial accelerometer (NXP Semiconductors; range ± 7G; sensitivity, 200 mV/G; size, 30 mm × 20 mm × 10 mm; mass, 13 g) was also placed over the center of the muscle belly at the right pectoralis major using double-sided adhesive medical tape. Movement across X, Y, and Z axes was sampled at 1000 Hz and collated using a root sum of squares in DasyLab™. Though only one MMG sensor was available for the current investigation, it was anticipated that a single sensor would be able to identify movement originating from other close muscle groups. For EMG and MMG assessment, diligence was paid with regards to the reproducibility of sensor placement in addition to the preparation of the skin, including removal of hair and cleaning in accordance with SENIAM recommendations (Hermens et al. 1999).

### Data analysis

Raw V̇O_2_, EMG, and MMG data outputs were subsampled into discrete 10 s average time blocks and graphed as a function of time in Microsoft Excel to synchronize sampling rates. Shivering onset was quantified using two independent methods for comparison, visual identification of data, and segmental regression. For every inflection point, the corresponding time elapsed (relative to entry into the bath) and *T*_re_ was also noted. *Visual Identification*: to determine intermittent shivering onset, a baseline value was first determined (a clear visual period of rest prior to shivering) and the inflection point is visually classified as short intermittent departure/s from baseline values, followed by a secondary inflection point for constant shivering without identifiable return to baseline (see Fig. 1 for example). In situations where individuals transitioned directly into constant shivering from baseline, the timepoint denoting both intermittent and constant shivering was recorded as the same timepoint. Visual identification of each inflection point was established independently by three members of the research team, with a median value calculated based on the three observations. Intraclass correlations (ICC) were calculated for this method of inflection point classification between researchers, observed to be, 0.969 for VO_2_, 0.994 for EMG, and 0.994 for MMG. *Segmental Regression:* two intersecting regression lines were added to each output using a least-squares fit, in which the point of intersect (*x*_0_) was not constrained. Once established, *x*_0_ was noted as the shivering inflection point. Using the segmental regression function in GraphPad software (Version 8; Prism, San Diego, USA), segmental regression was calculated as follows:

$$y_{1} = {\text{ intercept}}_{1} + {\text{ slope}}_{1} * \, x$$

$$y{\text{ at }}x_{0} = {\text{ slope}}_{1} * \, x_{0} + {\text{ intercept}}_{1}$$

$$y_{2} = \, y{\text{ at }}x_{0} + {\text{ slope}}_{2} * \, \left( {x \, {-} \, x_{0} } \right)$$

1 $$y \, = {\text{ IF }}\left( {_{X} < \, x_{0} , \, y_{1} , \, y_{2} } \right),$$

where *y*_1_ defines the intercept and slope of the first line segment, *y* at *x*_0_ is the *y* value of the first line segment when *x* = *x*_0_, and *y*_2_ computes the second regression segment from the *x*_0_ inflection point. The final line defines *y* for all values of *x*, where, if *x* <  × 0, then *y* = *y*_1_; otherwise, *y* = *y*_2_ (Motulsky 2016).

Comparisons between analytical inflection methods was made via Bland–Altman plot and ICC, based on an absolute agreement, two-way mixed-effects model (Koo and Li 2016). Segmental regression was independently compared to visually identified intermittent shivering (90 comparisons) and visually identified constant shivering (90 comparisons).

### Reliability and validity of metrics

Reliability statistics for comparisons between V̇O_2_, EMG, MMG and BSAS was undertaken as follows: mean coefficient of variation (CV) ± SD, assessing the within-subjects coefficient of variation across trials; ICC was assessed as above. Mean difference*,* assessing the within-subjects’ difference across trials was calculated as:

2 $$\mathrm{M}\mathrm{e}\mathrm{a}\mathrm{n} \mathrm{D}\mathrm{i}\mathrm{f}\mathrm{f} =\left(\frac{\begin{array}{c}\sqrt{{\left(T1-T2\right)}^{2}}+\sqrt{{\left(T2-T3\right)}^{2}}+\sqrt{{\left(T1-T3\right)}^{2}}\end{array}}{3}\right),$$

where *T*1, *T*2, and *T*3 are the *T*_re_ or shivering onset time in trial 1, 2, or 3*.*

Validity was then assessed in terms of the temporal comparison between V̇O_2_, EMG, MMG, and BSAS. The onset time was determined as the mean of the three trials and inflection point methods for each participant (Note, the authors also compared values for trial 1, 2, and 3 in isolation, showing similar conclusions). Differences between metrics were assessed via a paired samples *t* test. Statistical significance was set at *p* ≤ 0.05. Validity was also investigated using the signal-to-noise ratio (SNR ± SD) of each metric, highlighting the capacity for each metric to adequately detect shivering (signal) against background noise such as voluntary muscular movement, respiration, electrical noise, and vibration. The SNR for each metric was accounted for baseline noise (starting at the cessation of any initial cold shock response) of the 10 min period prior to shivering onset via segmental regression and compared this against the signal of the 5 min period post-shivering onset. The SNR was calculated as:

3 $$\mathrm{S}\mathrm{N}\mathrm{R}=\left(\frac{{( \stackrel{-}{x}}_{\mathrm{p}\mathrm{o}\mathrm{s}\mathrm{t}}-{\stackrel{-}{x}}_{\mathrm{p}\mathrm{r}\mathrm{e}} )}{{2\sigma }_{\mathrm{p}\mathrm{r}\mathrm{e}}}\right),$$

where ${\hspace{0.17em}\stackrel{-}{x}\hspace{0.17em}}_{\mathrm{p}\mathrm{r}\mathrm{e}}$ is the mean baseline value of the 10 min period prior to shivering onset, ${\stackrel{-}{x}}_{\mathrm{p}\mathrm{o}\mathrm{s}\mathrm{t}}$ is the mean shivering signal of the 5 min period post-shivering onset, ${2\upsigma }_{\mathrm{p}\mathrm{r}\mathrm{e}}$ is the two standard deviations of the variation in the baseline value of the 10 min period prior to shivering onset.

A ‘global SNR’ value was also generated, as the mean SNR of V̇O_2_, EMG, and MMG.

## Results

No statistically significant difference was seen in baseline *T*_re_ between trials at the point of cold water immersion and the start of the cooling phase (*p* = 0.7). An initial cold shock response was observed across most participants, subsequently settling after ~ 120 s and enabling individuals to remain still for the remainder of each trial. The cooling stimulus elicited shivering across all participants with an onset time ranging between 948 and 4409 s from initial immersion when comparing the mean of V̇O_2_, EMG, MMG, and BSAS measurements via segmental regression, visual inspection of intermittent, and constant shivering in any one given trial. The test–retest reliability of shivering onset across repeated trials (90 comparisons, again using the mean of all measures for each trial) was 0.90.

### Threshold identification: segmental regression vs. visual inspection

**Fig. 2:**
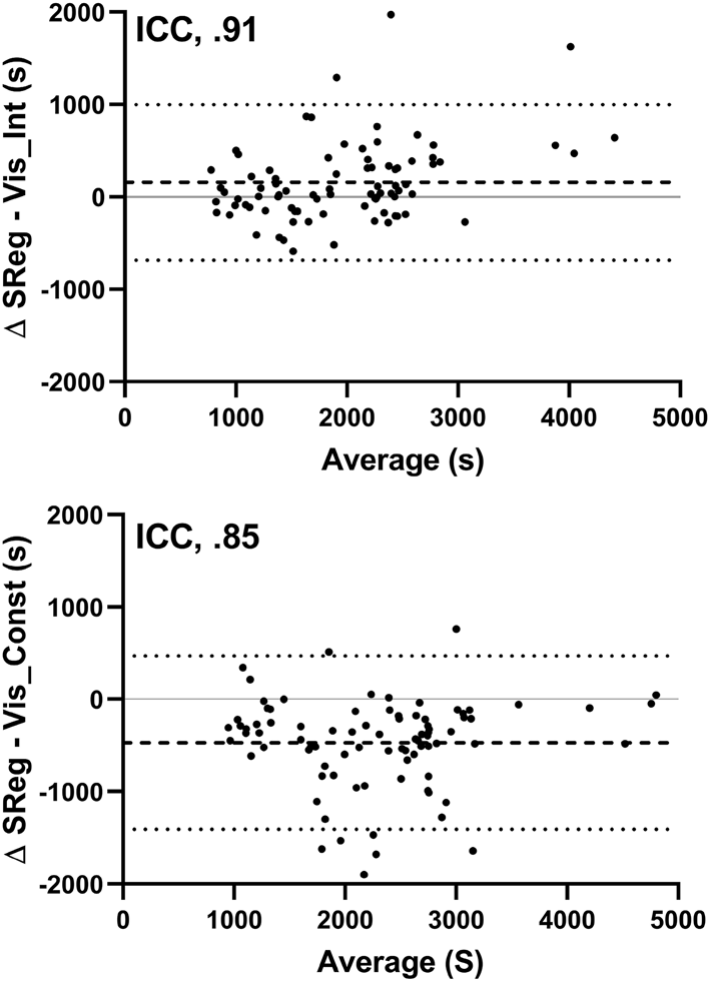
Bland–Altman comparisons between segmental regression and visual inspection for the identification of shivering thermogenesis onset. Segmental regression compared to visually identified intermittent shivering onset [TOP] and visually identified constant shivering onset [BOTTOM]. Ninety comparisons for each, accumulated from whole-body oxygen uptake, electromyography and mechanomyography measurements. Mean bias and limits of agreement are displayed

Comparing segmental regression with visual identification of inflections points (Fig. 2), a mean bias of 158 ± 430 s was observed with segmental regression following visually identified intermittent shivering. A mean bias of 471 ± 479 s was observed with segmental regression prior to visually identified constant shivering. Greater agreement was seen between segmental regression and visually identified intermittent shivering (ICC, 0.91), than was seen between segmental regression and visually identified constant shivering (ICC, 0.85).

### Test–rest reliability of V̇O_2_, EMG, MMG, and BSAS

Test–retest reliability data for measurement metrics are displayed in Table 1. In general, time provided a more consistent predictor of shivering onset than *T*_re_, (ICC’s; time, 0.73–0.96; *T*_re_, 0.78–0.86). All metrics presented good-to-excellent reliability [ICC’s > 0.75 and 0.90, respectively (Koo and Li 2016)], with the exception of visually identified intermittent shivering for V̇O_2_ measurement (ICC, 0.73) and segmental regression for EMG measurement (ICC, 0.74). MMG and BSAS marginally presented the most reliable indicators of shivering onset between trials, with the highest ICCs and the lowest mean difference between trials. This was closely followed by V̇O_2_ and EMG, which presented lower ICCs and higher mean differences (Table 1).

**Table 1:** Test–retest reliability of four independent metrics for the assessment of shivering onset during lower body cold water immersion, *n* = 10

	Trial 1	Trial 2	Trial 3	Mean diff	Mean CV (%)	ICC
**Onset time (S)**						
V̇O_2_						
*Vis_Int*	1847 ± 598	1851 ± 449	1966 ± 737	423 ± 344	15 ± 13	0.73
*Vis_Const*	2469 ± 640	2349 ± 683	2553 ± 871	413 ± 260	13 ± 7	0.88
*SReg*	1826 ± 754	1939 ± 754	1951 ± 861	364 ± 273	16 ± 11	0.88
EMG						
*Vis_Int*	1724 ± 835	2131 ± 986	2039 ± 968	402 ± 321	19 ± 15	0.91
*Vis_Const*	2429 ± 848	2625 ± 1121	2490 ± 893	482 ± 485	15 ± 14	0.88
*SReg*	1789 ± 686	2131 ± 986	2039 ± 968	537 ± 256	22 ± 13	0.74
MMG						
*Vis_Int*	2024 ± 703	2037 ± 945	1909 ± 744	314 ± 198	13 ± 8	0.95
*Vis_Const*	2665 ± 753	2795 ± 1083	2532 ± 1103	379 ± 277	12 ± 8	0.95
*SReg*	2249 ± 808	2091 ± 1077	2203 ± 1159	596 ± 440	21 ± 11	0.86
BSAS						
*Minor*	1728 ± 785	1689 ± 749	1555 ± 617	335 ± 244	15.1 ± 7.1	0.85
*Intermittent*	2352 ± 840	2421 ± 1054	2209 ± 845	359 ± 241	12.8 ± 8.4	0.95
*Constant*	2884 ± 870	2888 ± 1070	2714 ± 948	351 ± 217	10.0 ± 6.7	0.96
**Onset rectal temperature (°C)**						
V̇O_2_						
*Vis_Int*	37.00 ± 0.39	36.94 ± 0.58	37.04 ± 0.25	0.29 ± 0.18		0.79
*Vis_Const*	36.84 ± 0.41	36.68 ± 0.57	36.89 ± 0.22	0.30 ± 0.21		0.79
*SReg*	37.01 ± 0.39	36.85 ± 0.64	37.06 ± 0.25	0.30 ± 0.24		0.77
EMG						
*Vis_Int*	37.05 ± 0.39	36.95 ± 0.55	37.07 ± 0.23	0.28 ± 0.18		0.84
*Vis_Const*	36.83 ± 0.40	36.75 ± 0.58	36.00 ± 0.24	0.32 ± 0.21		0.78
*SReg*	36.94 ± 0.36	36.92 ± 0.54	36.93 ± 0.41	0.31 ± 0.15		0.80
MMG						
*Vis_Int*	37.03 ± 0.40	36.93 ± 0.54	37.13 ± 0.22	0.28 ± 0.19		0.80
*Vis_Const*	36.77 ± 0.43	36.67 ± 0.65	36.99 ± 0.22	0.35 ± 0.24		0.81
*SReg*	36.93 ± 0.38	36.91 ± 0.50	36.99 ± 0.23	0.26 ± 0.12		0.85
BSAS						
*Minor*	37.07 ± 0.38	37.03 ± 0.43	37.15 ± 0.21	0.26 ± 0.12		0.83
*Intermittent*	36.87 ± 0.41	36.79 ± 0.51	36.97 ± 0.24	0.28 ± 0.12		0.86
*Constant*	36.64 ± 0.40	36.63 ± 0.56	36.79 ± 0.21	0.27 ± 0.17		0.85

*V̇O*_*2*_ whole-body oxygen uptake, *EMG* electromyography, *MMG* mechanomyography, *BSAS* bedside shivering assessment scale, *Vis_Int* visual identification of intermittent shivering inflections, *Vis_Const* visual identification of constant shivering inflections, *SReg* Segmental regression, *Trial 1, 2 & 3* data are mean ± SD, *Mean Diff* mean ± SD of the within-subjects difference across trials, with a root sum of squares applied to ensure positive values, *Mean CV* mean ± SD of the within-subjects coefficient of variation across trials (inappropriate for rectal temperature data), *ICC* Intraclass correlation coefficient across trials, based on a mean rating (*k* = 3), absolute agreement, two-way mixed-effects model

### Validity of V̇O2, EMG, MMG, and BSAS

**Fig. 3:**
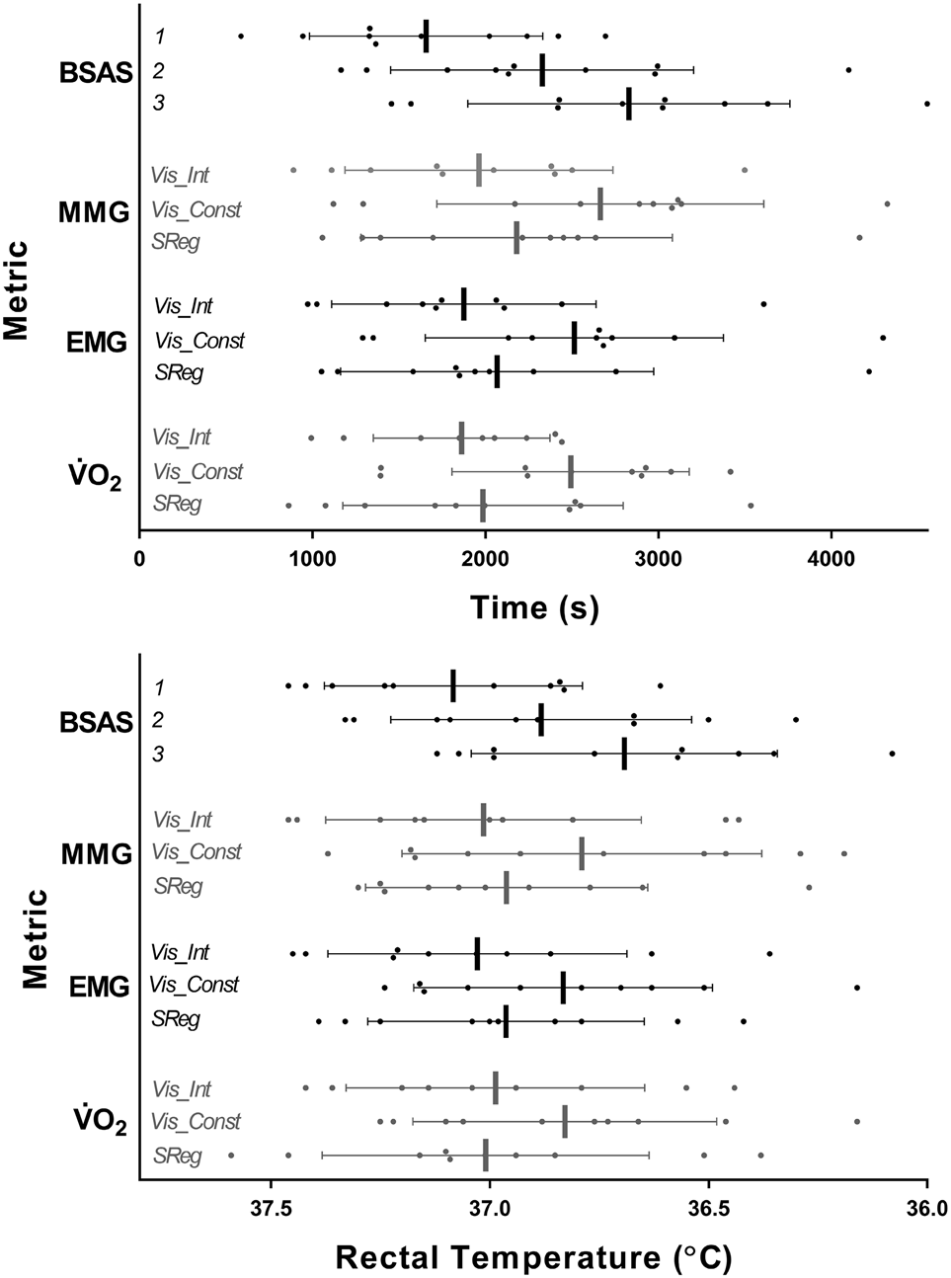
Onset of shivering assessed via various metrics during lower body cold water immersion, *n* = 10. Data are mean + SD, with individual data points, *n* = 10. Each data point represents the mean value of three trials for each participant. *V̇O*_2_ whole-body oxygen uptake, *EMG *electromyography, *MMG *mechanomyography, *BSAS* bedside shivering assessment scale, *Vis_Int *visual identification of intermittent shivering inflections, *Vis_Const *visual identification of constant shivering inflections. *SReg* Segmental regression

**Fig. 4:**
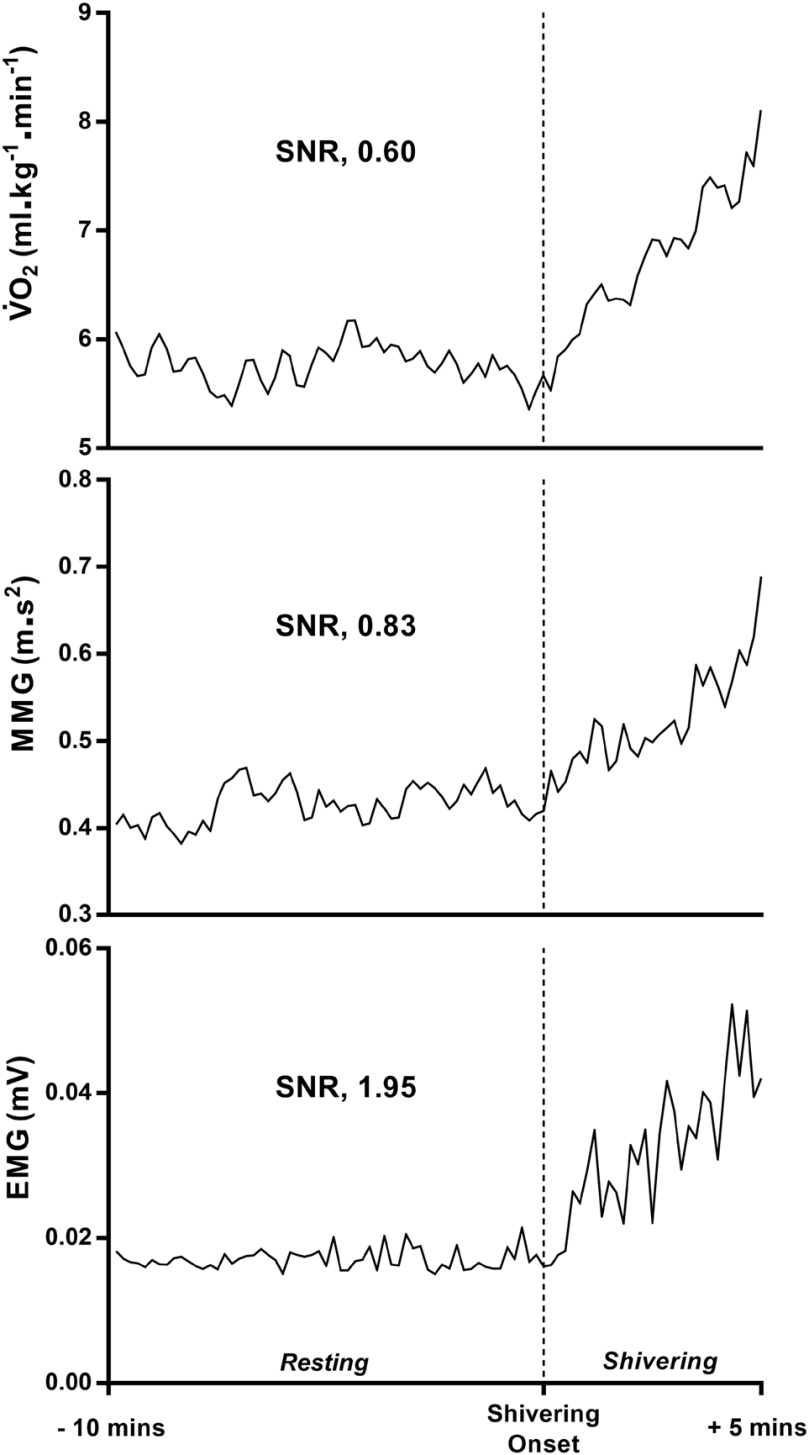
Schematic representation of the signal across three independent metrics in the assessment of shivering onset, *n* = 10. *V̇O*_2_ whole-body oxygen uptake, *EMG* electromyography, *MMG *mechanomyography. Data are means of three repeated trials across subjects. Shivering onset defined via segmental regression (least-squares fit)

Temporal comparison between metrics can be seen in Fig. 3, with a breakdown of infection points identified via segmental regression and visual inspection. Table 2 provides information on the mean bias between measurement metrics when segmental regression and visual inspection are combined for each. No correlation was observed between any specific anthropometric characteristics and adherence to this trend for earlier/delayed shivering identification when comparing metrics. In the assessment of signal-to-noise ratio, EMG showed the largest SNR at the point of shivering onset (SNR; 1.95 ± 4.07) followed by MMG (SNR; 0.83 ± 1.12) and finally V̇O_2_ (SNR; 0.60 ± 0.50). A visual example signal across metrics can be seen in Fig. 4. Combining SNR values for each metric into a mean produced a Global SNR of 1.13 ± 0.72.

**Table 2:** Comparison between four metrics in the measurement of shivering onset with cold water immersion, *n* = 10

Shivering onset metric	Mean bias	
	Time (s)	*T*_rec_ (°C)
V̇O_2_–EMG	42 ± 336	0.01 ± 0.06
V̇O_2_–MMG	− 82 ± 234	0.01 ± 0.07
V̇O_2_–BSAS	− 99 ± 242	0.06 ± 0.09*
EMG–MMG	− 124 ± 194	0.01 ± 0.06
EMG–BSAS	− 141 ± 315	0.06 ± 0.10
MMG–BSAS	− 17 ± 240	0.05 ± 0.12

Data are mean ± SD obtained from three repeated trials. *V̇O*_*2*_ whole-body oxygen uptake. ***EMG,*** electromyography, *MMG *mechanomyography, *BSAS *bedside shivering assessment scale. *Significant difference, *p* < 0.05

## Discussion

This study examined the reliability and validity of two independent analytical methods used to establish the shivering thermogenesis inflection/break point (segmental regression and visual inspection) and four independent measurement metrics in the assessment of the shivering thermogenesis (V̇O_2_, EMG, MMG, and BSAS). Discussion points are made herein with reference to guidelines defining ICC values greater than 0.75 as expressing good reliability and ICCs greater than 0.90 as expressing excellent reliability (Koo and Li 2016). Primary findings include: first, the data from this investigation show shivering onset between repeated visits with the same cooling stimulus to be highly reliable. Second, segmental regression provides an appropriate method for assessing shivering onset, showing excellent agreement with visual inspection of inflection points by three independent researchers. Finally, comparing shivering measurement metrics, no single metric provided a superior method of onset identification relative to the others; all metrics scored well, yet subtle differences were observed in accordance with reliability, temporal comparison, and the signal-to-noise ratio.

### Estimating the shivering threshold: segmental regression vs. visual identification

Previous research has successfully validated the use of segmental regression against a variety of rater methods in the estimation of the sweating threshold (Cheuvront et al. 2009). Despite the potential for many confounding factors to distort a ‘clean’ inflection point in the onset of shivering thermogenesis (i.e. voluntary muscular activation), the current study also shows excellent agreement between estimates of shivering onset via segmental regression with visual inspection of inflection points. In this study, independent raters were asked to distinguish between intermittent and constant shivering inflection points where possible to compare with segmental regression. The data suggest segmental regression estimation of shivering onset to temporally agree better with visually identified intermittent shivering onset than with constant shivering onset. The extent to which this agreement is altered with different cooling protocols, where the ratio of intermittent shivering to constant shivering duration is possibly altered, has yet to be investigated. Furthermore, the inability of segmental regression to distinguish intermittent and constant shivering remains as a conceivable limitation in the comparison between the two methods.

### Test–retest reliability of V̇O_2_, EMG, MMG, and BSAS

In view of the ICC values observed in the current study, all four metrics provide a good-to-excellent degree of test–retest reliability in the assessment of shivering onset. Though all metrics scored well, the data suggest MMG and BSAS to be slightly more reliable for use in repeated measures studies. Note, the current study utilized lower body water immersion in which the skin temperature of the lower body was fixed close to water temperature. Given that skin temperature provides one of several affector inputs to shivering onset, the reliability of metrics under situations where skin temperature is less constrained that is yet to be established, for example, during cooling with air or a water-perfused suit. Interestingly, time elapsed relative to entry in the bath provided a more consistent predictor of shivering onset than core temperature. This finding may challenge the exclusive role of central and peripheral thermal receptors, and thereby set point theory in the onset of cold-induced thermoeffector activity, providing partial evidence for the contribution of non-thermal factors, including the input of a temporal factor. Indeed, during initial exposure to a cold stimulus, it is conceivable that temporal aspects of discomfort and anxiety trigger various anticipatory thermoeffector responses, prior to the establishment of expected heat-balance responses as seen purely through a biophysical model. This point may be further emphasized given the fact that skin temperature was again well controlled for in the current study, lower body skin temperature clamped at the temperature of the water, thus if a core-temperature set point was the sole trigger for shivering thermoeffector responses, a fixed *T*_re_ should have been observed irrespective of the cooling rate and the pre-immersion body temperature. With this in mind, the sensitivity of rectal temperature, as used in this study, to track minor changes in core temperature should be considered.

### Validity of V̇O_2_, EMG, MMG, and BSAS

Thermal research and clinical practice assessing real-time shivering responses necessitate metrics that are capable of providing instantaneous data feedback, hence temporal comparison of shivering onset metrics may be of interest. Previous research has indicated the potential for MMG to identify mild shivering with improved sensitivity over EMG (McKay et al. 2013). As such, we conjectured that MMG would detect shivering onset marginally earlier than EMG, with a reasonable delay in shivering onset detected via V̇O_2_ assessment due to the temporal nature of O_2_ kinetics. Data from this investigation show no statistically significant temporal difference between measurement metrics, yet several interesting differences (Fig. 3*,* Table 2). In disagreement with our conjecture, EMG and V̇O_2_ were able to identify shivering onset earlier than the MMG. This observation could be due to the use and availability of a single MMG sensor in the current investigation. Yet, this MMG sensor would conceivably has the capacity to register mechanical movement from other surrounding muscle groups upon which EMG sensors were additionally placed. Furthermore, when comparing MMG onset time to that of the EMG from the pectoralis major alone, a lag in MMG identification was still observed behind EMG (162 ± 253 s). The earlier than expected detection of shivering onset via V̇O_2_ may be due to its capacity to detect thermogenic activity from the lower body, which would have been missed by EMG and MMG recordings. In the current investigation, the opportunity to measure EMG and MMG activity in the lower body was inhibited by water immersion.

Interestingly, BSAS stage 1 (minor localised shivering-related twitches) identified shivering-related activity prior to any detection via objective metrics. This finding contrasts previous research indicating that EMG and MMG can identify shivering activity prior to visual detection (Hemingway 1963; McKay et al. 2013). However, this finding is less surprising given the fact that visual identification assessed whole-body muscular activity, including areas of the body submerged in water at any one time, while EMG and MMG could only detect muscular activity at the measured muscle group(s). This may have led to early shivering-related activity being missed as a result of variations in the order of activated muscle groups at the point of shivering onset (Haman and Blondin 2017). Yet, it is also important to recognize that the definition of BSAS stage 1 used in the current investigation is broad and may only be used to indicate the presence of an imminent shivering response, rather than a robust physiological entity. In clinical practice, BSAS would also require a clinician/nurse to observe a patient constantly. Furthermore, though each BSAS stage is designed to focus exclusively on shivering thermogenesis, it is possible that at very mild shivering intensities, the assessor responds to and subconsciously integrates behavioral cues (i.e. minor fidgeting and signs of discomfort) into their final scoring.

### Integration of metrics into a live measure

The current investigation provides evidence for the simple and convenient use of segmental regression in determining shivering onset, yet a drawback of such method remains that it can only be completed as a post-test analysis. A progression of the current investigation is the development of a live integrated measure of shivering onset, which would enhance the way that non-thermal factors can be implemented and addressed in cold focused research. It is evident that each metric in isolation presents a series of key limitations, thus the alignment of three independent objective metrics V̇O_2_, EMG and MMG, into a single collective ‘global’ measure, could serve to enhance both confidence and precision in defining the onset of shivering. Furthermore, real-time calculation of SNR data provides a possible medium for live shivering onset assessment. Equation 3 can be adopted to provide a rolling metric for any given timepoint of each metric in which a spike in signal would indicate an inflection point. Equation 4 provides an example of such, in which a rolling 5 min sampling period is used for the generation of a ‘signal’ value and compared to a rolling 10 min sampling period for the generation of a ‘noise’ value.

4 $${\rm{SN}}{{\rm{R}}_{{\rm{live}}}} = \left( {\frac{{\left( {\sum _{t = 0}^{t = - 300}/300 - \sum _{t = - 300}^{t = - 900}/600} \right)}}{{2{\sigma _{\left( {t = - 900\,to\,t = - 300} \right)}}}}} \right),$$

where SNR_live_ is a real-time rolling SNR for each metric, *t* is the current time elapsed in seconds, 2σ is two standard deviations.

Based on the results of the current study, shivering onset would be classified by a Global SNR (the mean SNR_live_ of V̇O_2_, EMG, and MMG data) greater than 1.13. It should be noted that this definition attributes equal weighting to each of the three metrics in the determination of Global SNR, while future work could seek to establish whether or a not a weighting factor would be required for each metric. Furthermore, to address subtle temporal differences between metrics, the introduction of logic-based decision criteria would reduce the chances of shivering onset being missed or delayed through a global measure. For example, if two of the three metrics in isolation (i.e. EMG, and MMG) were to reach their respective magnitude increase in SNR without cross validation of the other metric (i.e. V̇O_2_), this could also be classified as onset. Further research on such integrated measure would increase the translatability of shivering work across institutes.

## Conclusion

This investigation validates the use of segmental regression in determining shivering onset. Comparing V̇O_2_, EMG, MMG, and BSAS, good-to-excellent reliability can be seen across all metrics, yet given the observed lag times, signal-to-noise ratios, along with known advantages/disadvantages of each metric, multiple metrics should be used in shivering measurement. The results from the current investigation can be used to develop a live integrated multi-modal measure of shivering, with an initial framework of such presented herein.

## Abbreviations

BSAS: Bedside shivering assessment scale
CI: Confidence interval
EMG: Electromyography
ICC: Intraclass correlation
MMG: Mechanomyography
SNR: Signal-to-noise ratio
SReg: Segmental regression
*T*_re_: Rectal temperature
Vis_Int/Vis_Const: Visually identified intermittent/constant shivering inflections
V̇O_2_: Oxygen uptake
